# Spatial Compression Impairs Prism Adaptation in Healthy Individuals

**DOI:** 10.3389/fnhum.2013.00165

**Published:** 2013-05-07

**Authors:** Rachel J. Scriven, Roger Newport

**Affiliations:** ^1^School of Psychology, University of NottinghamNottingham, UK

**Keywords:** neglect, PA, spatial compression, MIRAGE mediated reality, prism aftereffects, strategic motor control, error-detection threshold

## Abstract

Neglect patients typically present with gross inattention to one side of space following damage to the contralateral hemisphere. While prism-adaptation (PA) is effective in ameliorating some neglect behaviors, the mechanisms involved and their relationship to neglect remain unclear. Recent studies have shown that conscious strategic control (SC) processes in PA may be impaired in neglect patients, who are also reported to show extraordinarily long aftereffects compared to healthy participants. Determining the underlying cause of these effects may be the key to understanding therapeutic benefits. Alternative accounts suggest that reduced SC might result from a failure to detect prism-induced reaching errors properly either because (a) the size of the error is underestimated in compressed visual space or (b) pathologically increased error-detection thresholds reduce the requirement for error correction. The purpose of this study was to model these two alternatives in healthy participants and to examine whether SC and subsequent aftereffects were abnormal compared to standard PA. Each participant completed three PA procedures within a MIRAGE mediated reality environment with direction errors recorded before, during and after adaptation. During PA, visual feedback of the reach could be compressed, perturbed by noise, or represented veridically. Compressed visual space significantly reduced SC and aftereffects compared to control and noise conditions. These results support recent observations in neglect patients, suggesting that a distortion of spatial representation may successfully model neglect and explain neglect performance while adapting to prisms.

## Introduction

Neglect syndrome is typified by an inability to explore, or react to objects and events in, the side of space contralateral to a cerebral lesion (Halligan and Marshall, 1993) and is most commonly associated with right hemisphere strokes (Halligan et al., 1990). Unilateral neglect is far more common on the left, following right hemisphere lesions, than right neglect, following left-hemisphere lesions (Corbetta et al., 2005). It is distinct from primary sensory and motor deficits as demonstrated by behavioral testing. Lesion sites do not necessarily include primary regions (Heilman et al., 2003) and spontaneous recovery is faster than that which follows primary damage (Halligan and Marshall, 1993). It presents as a very heterogeneous disorder, with various subcomponents depending on lesion site and extent of damage (e.g., Buxbaum et al., 2004; Verdon et al., 2010).

Neglect patients present with a range of related behaviors, such as colliding with objects on the left, attending only to the right side of their body, and eating only the left half of a plate of food, losing objects, and failing to respond to people in the neglected space. They often have difficulties reading, writing, and drawing, and even remembering the left half of a familiar memory or scene (Bisiach and Luzzatti, 1978; Wilson, 1999). Patients also commonly lack insight into their condition, which significantly influences rehabilitation progress (Kinsella and Ford, 1985; Appelros et al., 2002). As such neglect has significant clinical implications, with a severe effect on daily function.

While a number of rehabilitation therapies have been developed and tested (Bowen and Lincoln, 2007), PA has been found to be one of the more effective, long-term, and simple strategies. Rossetti et al. (1998) demonstrated that a PA procedure significantly reduced neglect behaviors in classic tests including line bisection, cancelation, drawing, and reading for up to 2 h, significantly longer than 10-min effects in previous methods. Prism goggles cause a shift in visual input relative to the proprioceptively defined position of the limb, resulting in individuals mis-reaching in the direction of the prismatic shift when trying to point to or grasp a target. PA occurs when participants quickly learn to adjust their reach to become accurate again. After a short but sufficient training period, when the prism goggles are removed participants will mis-reach in the direction opposite to prismatic shift. These *aftereffects* reflect the recalibration of reference frames for visuo-motor maps in order to realign them (Redding and Wallace, 2006).

While it is possible that the aftereffects merely neutralize the neglect bias due to a contraversive shift, the fact that PA improves performance in attentional and perceptual tasks, as well as visuo-motor tasks, indicates a genuine improvement in neglect behaviors (Newport and Schenk, 2012). Stable effects are shown to generalize across a range of neglect behaviors including postural control, tactile, and auditory extinction, mental imagery (Rode et al., 2001), number line bisection, neglect dyslexia, oculomotor biases, and even wheelchair navigation (Arene and Hillis, 2007). An additional benefit as a rehabilitation technique is that PA is a bottom-up technique and does not require an awareness of the disorder. In a review by Newport and Schenk (2012), more than 90% of studies found a positive effect of PA in reducing neglect so long as the prismatic shift was strong enough and included repeated treatment sessions for long-term effects. Indeed, a recent study reported permanent improvements following long-term daily PA treatment (Nijboer et al., 2011).

It is likely that PA may not affect all neglect component behaviors (e.g., Striemer and Danckert, 2010; Fortis et al., 2011a,b), but may be valuable in identifying a meaningful subcomponent of neglect and its underlying pathology. The unique relationship between PA mechanisms and neglect syndrome is both important and unclear, and further investigation may provide a novel theoretical framework on which to focus new lines of research. Two primary mechanisms, “SC” and “spatial realignment” (SR), have been identified during the realignment of visuo-motor systems in PA. These processes dissociate (Pisella et al., 2004; Newport and Jackson, 2006; Aimola et al., 2012) and are comprehensively addressed by Redding and Wallace (2006), but will be briefly detailed here. Initial corrections for prism-induced errors can be made on-line during the reaching movement, or in subsequent movements by deliberately mis-reaching in the direction opposite to the prismatic deviation. This “SC” is a rapid and conscious process, and is useful for remapping spatially coded movement commands in a dynamic environment in order to reduce performance error (Redding and Wallace, 2006). However, SC is not sufficient for aftereffects to occur and a greater number of trials are required for the second, slower process of “SR.” SR is an unconscious recalibration of visual and motor co-ordinate systems used to plan goal-directed actions, as a result of which, when the prisms are removed after sufficient trials participants now miss in the direction opposite to the prismatic shift. After prism removal, with continued pointing to visual targets, healthy participants are typically very fast to deadapt and return to baseline accuracy (see Redding and Wallace, 2006; Newport and Schenk, 2012 for more detailed explanations of these processes).

These mechanisms do not simply counter the neglect bias since they do not account for the remarkably long-term effects of PA specifically found in neglect patients, which are significantly longer than comparable stimulation techniques (Rossetti et al., 1998). It has been suggested that abnormally long-lasting aftereffects may be due to a reduced awareness of prism-induced errors. Redding and Wallace (2006) proposed that in healthy individuals SC may limit the need for SR, and consequently, a dysfunctional SC may remove this limit leading to extraordinarily larger aftereffects. Michel et al. (2007) supported this idea, citing anecdotal evidence for neglect patients having reduced awareness of visual perturbations caused by prism goggles from studies by Rode et al. (2003) as well as their own investigations of unaware PA in healthy controls. If error awareness is a precondition for SC, this “hypernosognosia” – over-self-attribution of movement error – may lead to an increased dependency on SR processes. They found evidence to support this by incrementally increasing the prism shift in healthy individuals, with reduced error awareness of PA resulting in larger aftereffects. Aimola et al. (2012) tested this idea in neglect patients and confirmed reduced SC in neglect, with patients showing significantly less adaptation than right-brain damaged controls and healthy controls, failing to eliminate prism-induced error even after 72 reaches. However, they also found that aftereffects were not pathologically increased, contradictory to predictions. While proprioceptive aftereffects are often considered key to neglect recovery following PA (e.g., Sarri et al., 2008; Fortis et al., 2010, 2013), others argue that they dissociate from the persistence of neglect amelioration and that it is the adaptive processes involving SC which are predictive of recovery (e.g., Serino et al., 2006; Ladavas et al., 2011).

Aimola et al. (2012) suggested that poor SC in neglect might be caused by dysfunctional error-detection, either due to a pathological failure to detect errors for which the error signal falls in neglected space or, alternatively, that there is an increased tendency to treat reaches with errors as being under the patient’s own control (hypernosognosia). In both cases, deliberate inter-trial error correction would be unnecessary: in the former, there are no errors to correct and in the latter the strategic correction of sub-threshold errors would not be required. On the one hand errors are simply not detected, while on the other errors may be detected, but are treated as being within normal limits. In order to investigate this further, the current experiment was designed to measure the effects of introducing environments that encouraged each of these potential causes for dysfunctional error correction in healthy controls during a PA task. A failure to detect errors was modeled by compressing visual space such that errors were perceived as much smaller than in reality and hyponosognosia was modeled by introducing small visual perturbations, or noise, to the motor output in order to blur the boundaries between reaches that were self-generated and those that were as a result of the prism displacement and therefore requiring strategic correction. Both of these ideas will be expanded upon in the next sections.

Typically, error-detection and correction involves neural comparator mechanisms which detect discrepancies, such as between the intended outcome of a movement and the predicted or (estimated) actual outcome of that movement (Wolpert, 1997). Small errors result in largely unconscious movement correction while larger errors can lead to the attribution of movement control to an external agent or influence. In the case of prism-induced errors, this would lead to the deliberate and strategic correction of movement parameters in subsequent reaches. Dysfunctional processes in neglect might lead to impaired error-detection either by damage to neural comparators or by interrupting or distorting input to the comparator system. The failure of movement discrepancies to reach conscious thresholds would remove the requirement to correct movement errors on subsequent trials and also to an over-attribution of erroneous movements as being judged as self-generated (i.e., not as a consequence of wearing prisms).

Hypernosognosia, the over-self-attribution of movement agency, was observed in a small group of neglect patients by Preston et al. (2010) who found that they exhibited an over-attribution of self-generated movement in line with that suggested by Michel et al. (2007). In that study, patients gripped a mechanical arm with their unaffected hand while making goal-directed reaches, but the computer-generated visual feedback of the movement was perturbed to the left or right to varying degrees on a trial-by-trial basis. Neglect patients were poorer at detecting modifications to their own movements, tending to self-attribute reaches at larger perturbations than controls, while being better at the task than a patient with anosognosia for hemiplegia. The authors postulated that the comparators typically responsible for detecting errors may be damaged or have raised thresholds in neglect patients, and so do not consciously register an error. If this is the case then it could be modeled in healthy controls by introducing “noise” to their movements by giving visual feedback with perturbations at close-to-threshold limits so that reaches consistently miss slightly to the left or right. The introduction of noise would potentially raise intact comparator error-thresholds, resulting in greater self-attribution of errors and reduced SC.

The alternative mechanism, one in which errors are not detected, is less straightforward. Aimola et al.’s (2012) proposal was that the failure to detect errors was specific to rightward errors; that is, those in which the target falls to the left of the hand as it does during the early stages of rightward PA. Their proposal was that the target error, being to the left of the hand, falls in neglected space (or, at least, space that is more compressed than the space to the right of the hand). However, with targets in PA often being spread across the workspace, it is not certain whether the error would necessarily fall in neglected space or even whether patients look toward the target or the hand (or both) when the hand becomes visible toward the end of the reach. A potential answer to this problem might be to create a workspace that is modeled on the spatial compression theory of neglect. By using this model, it would not matter whether the patient fixates the hand or the target because the separation between the two would be perceived to be smaller (compressed) compared to reality.

Halligan and Marshall (1991) proposed a left-to-right compression of space based on a neglect patient’s systematic deflection in judgments of target position. Keller et al. (2000), who also found evidence in accordance with neglect patients’ distorted egocentric representation, proposed that this results from the dynamic remapping of space based on imbalanced input. An attentional distribution may cause such an imbalance, leading to a compression of the affected hemi-space relative to ipsilesional space. Kerkhoff (2000) found distortions of perceived space between objects and both Kerkhoff (2000) and Harvey et al. (2007) observed misrepresentations of object size in the horizontal plane in accordance with theories of anisotropic representation of space in which only the horizontal dimension of visuo-spatial representations might be relaxed toward contralesional and compressed toward ipsilesional space in accordance with Bisiach et al. (1998). It has been argued that such compression has also been observed during reaching tasks: Jackson et al. (2000) found evidence for a distorted topography of representation in neglect, revealed by abnormally curved hand paths to visually defined targets compared to proprioceptively defined targets, indicative of an impairment in the visual space used to guide movements, without a general failure of the spatial representation of target position.

In a hypothetical representation of space in which the dynamic workspace to the left of the hand is compressed, both the hand and target would be visible at the end of the prism-displaced reach, but the distance between the two would be perceived to be smaller than in reality preventing the efficient detection of reach errors. Such compression would also allow for the direction-specific effects described by Aimola et al. (2012) in which hand-target errors to the right of the hand are detected normally whereas errors to the left are not. The hypothesis here is that compressed space would prevent the detection of errors that are specifically to the left of the hand, hindering SC during adaptation, but not during deadaptation when the error would fall to the right.

The present study aimed to investigate these two competing theories in relation to PA by modeling them in healthy individuals. A typical PA procedure was employed in which participants completed reaching movement toward visual targets before, during, and after PA in the two modeled neglect conditions and a control condition. By comparing the pattern of PA between these conditions, it can be examined whether they successfully impair SC as suggested by Aimola et al. (2012) and also any consequent dissociation of SC and SR in these conditions.

## Materials and Methods

### Participants

Twelve participants (11 female; mean age 21 years, range 18–25) took part in the study as volunteers. All were healthy, right-handed undergraduate students with normal or corrected-to-normal vision. All participants gave informed consent and the experiment was conducted in accordance with the Ethics Committee at the University of Nottingham.

### Apparatus and stimuli

**Figure 1:**
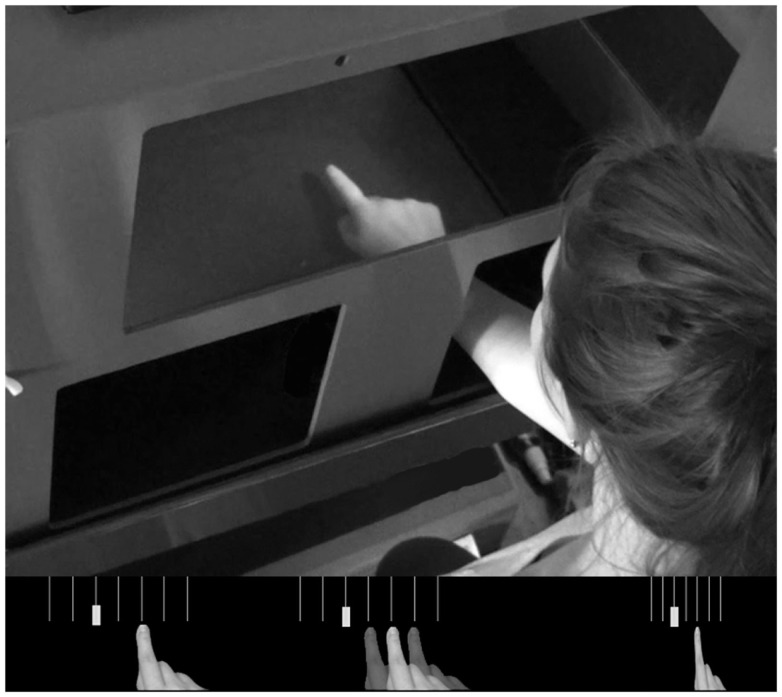
**Top panel; the MIRAGE mediated reality device used throughout the experiment**. MIRAGE modifies real-time video capture of the real limb and displays it in the same plane as the actual limb. Bottom panel: a schematic representation of each condition. Left – Control; middle – Noise; right – Compression. Semi-opaque hands represent the addition of left and right noise perturbations. Vertical lines represent 3° separations in real space (not visible to participants). In each panel, the solid hand represents a real space reaching error of 6°.

The entire experiment was conducted using a MIRAGE mediated reality device (Newport et al., 2010) in order to create the various visual feedback conditions. MIRAGE uses cameras and mirrors to display a live (delay ∼20 ms) video image of the participant’s own hand in the same physical location as their real hand (see Figure 1). Although the real hand is never seen directly, participants treat the representation as their own hand without a noticeable delay (e.g., Newport and Preston, 2010). Perturbations to the visual feedback presented to the participant were calculated on-line and involved displacement-dependent lateral shifts of the viewed image of the hand based on the moment-to-moment location of the real hand. The location of the real hand and the targets were recorded and monitored on-line using a Polhemus Liberty electromagnetic motion tracker sampling at 60 Hz. Single Polhemus sensors were attached to the nail of the right index finger and to both targets. For conditions which required the location of the hand to be hidden from the participant for some or all of the movement, this was achieved by replacing the relevant pixels in the image with a zero value, creating the illusion of a virtual bar across the workspace.

The two targets (physical objects seen within the MIRAGE environment) were placed 20 cm forward and 2.5 cm to the left and right of a tactile start point placed close to the leading edge of the workspace and 7.5 cm to the right of the midline. For each trial only one target was visible, displayed in a pseudorandom order such that no target appeared three times in succession, with the other being removed from the image digitally. For the adaptation phases of the experiment, participants wore base-left 10-diopter wedge prisms, deviating vision by ∼6° to the right. While 10-diopter prisms are relatively weak in terms of neglect research (see Newport and Schenk, 2012), their use here was both necessary and appropriate due to a combination of the close confines of the MIRAGE apparatus and the magnitude of the deviation applied in the Noise condition to which the other conditions were compared.

### Procedure

For each condition there were six phases completed in a set order (see Table 1) involving two pre- and post-test measurements either side of the experimental adaptation condition and a final deadaptation phase. Pre-Adaptation Open Loop (PreOL) involved pointing to each target twice without visual feedback of the hand. This was taken as the baseline against which Post-Adaptation Open Loop (PosOL) pointing was compared in order to assess the magnitude of prism-induced aftereffects. The procedure for PosOL was identical to that for PreOL. Pre-Adaptation Visual Feedback (PreVF) involved pointing twice to each target with terminal visual feedback of the limb (terminal visual feedback refers to the hand only being visible toward the end of the reach – on this occasion, the last 20% of movement distance). PreVF was the baseline against which post-adaptation accuracy (PosVF) was measured with the procedure for PosVF being identical to PreVF. To avoid open loop measures being tainted by exposure to vision of the hand, PreOL, and PosOL always preceded PreVF and PosVF respectively. Between the pre- and post-accuracy measures, participants wore 10-diopter prism goggles and pointed 40 times (20 to each target) in one of three PA conditions. In the Control condition (standard PA), visual feedback was an accurate representation of the actual reach. In the Noise condition visual feedback was perturbed such that reaches were shifted by 3° to the left or right of the actual hand path in a pseudorandom order such that no particular perturbation could be presented three times in succession. Three degrees was chosen as a recent experiment using similar equipment, but investigating attribution of movement agency, revealed that participants were below chance when judging whether movements with 3–4° perturbations were self-generated (that is, more often erroneously rating them as self-generated when they were not) (Preston and Newport, 2010). For the Compression condition a simple spatial compression was applied to the visual workspace such that everything was compressed to the right. This was achieved by removing every alternate vertical line of pixels from the displayed image of the workspace. Thus, objects (such as the targets) to the left of the workspace were compressed rightwards by a greater degree than those toward the right of the workspace. For example: in a hypothetical workspace 20 cm wide, an object on the left hand edge, 20 cm from the right edge, would be compressed to appear 10 cm (20/2 cm) to the right of its real location; an object in the center, 10 cm from the right edge, would be compressed 5 cm rightwards (10/2 cm) and an object 5 cm from the right hand edge would be compressed 2.5 cm (5/2 cm) rightwards. The functional effect of the compression was that of halving the apparent magnitude of any directional reaching error. Finally, a further 26 deadaptation reaches were made, with full visual feedback, in order to return the participant to normal levels of pointing accuracy in preparation for the next condition. Participants carried out all three PA conditions in a counterbalanced order between participants.

**Table 1:** **Phase order and number of trials per phase with visual feedback conditions**.

Phase	Trials	Visual feedback
Pre-open loop (PreOL)	4	No visual feedback of the hand
Pre-visual feedback (PreVF)	4	Terminal visual feedback
Prism-adaptation (PA)	40	Terminal feedback
Post-open loop (PosOL)	4	No visual feedback of the hand
Post-visual feedback (PosVF)	4	Terminal visual feedback
Deadaptation	26	Terminal visual feedback

## Results

Reach errors were calculated as the difference in degrees between straight lines from the start point to the target and the start point to the index finger at the end of the reach. In order to remove late movement corrections based on visual feedback of the hand, movement end-point was determined by the movement frame in which the finger would have become visible (i.e., breaching an imaginary line 4 cm short of the target distance). Thus, any reduction in reach end-point errors would have been the result of both adaptation and inter-trial strategic correction, but would have excluded on-line within-trial conscious error reduction. For analysis, trials were binned into target pairs so that each data point represented the mean of a reach to both a left and a right target.

### Adaptation phase

**Figure 2:**
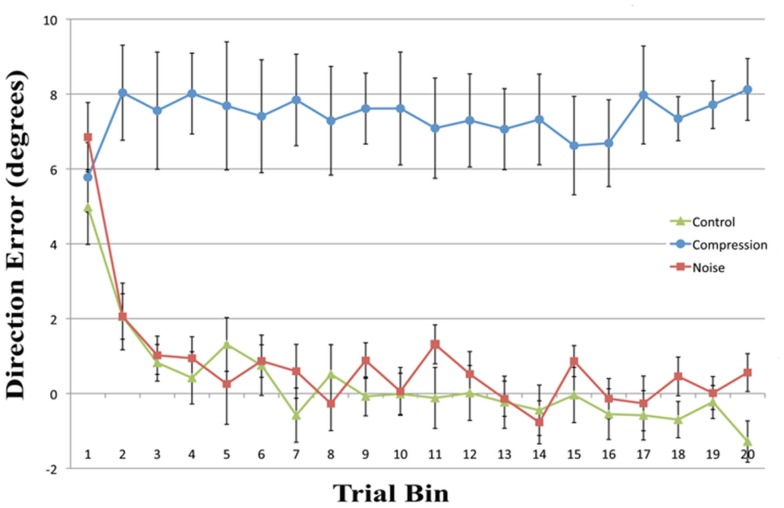
**Mean directional pointing error (with SE bars) for each two-trial bin in the prism-adaptation phase for all three conditions**. Positive values indicate a rightwars error in the direction of the prism displacement.

Mean end-point error for the first four bins and the final bin were entered in a two-way repeated measures ANOVA with the factors CONDITION (Noise, Control, and Compression) and BIN (One, Two, Three, and Four). The analyses revealed a significant main effect for CONDITION [*F*(2, 22) = 128.1, *p* < 0.001] and BIN [*F*(4, 44) = 13.6, *p* < 0.001] as well as a significant interaction [*F*(8, 88) = 5.0, *p* < 0.01]. In order to assess the rate and ultimate success of error correction, planned pair-wise comparisons were conducted between each condition pair for the first four bins and the last bin with the alpha level corrected to 0.0033 for multiple comparisons. While there were no differences in accuracy between any of the conditions for the first bin [Max: *F*(2, 22) = 2.33, *p* = 0.13], the Compression condition was significantly less accurate than either the Control or Noise condition for bins 2–4 and bin 20 [Min: *F*(2, 22) = 26.10, *p* < 0.001] while the latter two conditions were not different from each other in any bin [Max: *F*(2, 22) = 2.26, *p* = 0.14]. In short, while both Noise and Control showed normal PA error reduction, reducing rapidly to baseline accuracy, participants failed to adapt in the Compression condition, even after 40 trials (see Figure 2).

### Aftereffect

Mean end-point errors for the first bin in the open loop trials were also entered in a two-way repeated measures ANOVA with the factors CONDITION (Noise, Control, and Compression) and PHASE (Pre-adaptation, post-adaptation). The analyses revealed a significant main effect for PHASE [*F*(1, 11) = 24.0, *p* < 0.001], but not CONDITION [*F*(2, 22) = 2.2, *p* > 0.05] although there was a significant interaction [*F*(2, 22) = 17.2, *p* < 0.001]. Planned pair-wise comparisons were run between “preOL” and “posOL” trial bins to determine whether adaptation had occurred for each condition (see Table 2). There was a significant difference between “PreOL” and “PosOL” in the Control [*F*(1, 11) = 23.1, *p* < 0.001] and Noise conditions [*F*(1, 11) = 53.7, *p* < 0.001] with “PosOL” having a greater leftward error in both conditions, but there was no significant difference between “PreOL” and “PosOL” for the Compression condition [*F*(1, 11) = 0.59, *p* = 0.45] indicating an absence of aftereffects following the adaptation phase.

**Table 2:** **Mean (with SD) directional pointing error in degrees for the first four trials in each phase in the Control, Compression, and Noise conditions**.

	Pre-open loop	Pre-visual feedback	Prism-adaptation	Post-open loop	Post-visual feedback
Control	−1.74 (4.36)	−0.59 (4.06)	2.06 (3.02)	−7.83 (4.74)	−3.68 (2.81)
Compression	−1.95 (5.31)	−2.32 (3.61)	8.66 (4.29)	−2.27 (4.51)	−1.56 (1.89)
Noise	−0.21 (4.07)	−0.35 (3.60)	2.71 (3.50)	−8.34 (4.82)	−3.41 (3.04)

*Negative values indicate a leftward error in the direction opposite to the prism displacement*.

### Deadaptation

As with Adaptation measures, mean end-point error for the first four bins and the final bin were entered in a two-way repeated measures ANOVA with the factors CONDITION (Noise, Control, and Compression) and BIN (One, Two, Three, and Four). The analyses revealed a significant main effect for CONDITION [*F*(2, 22) = 8.9, *p* < 0.05] and BIN [*F*(4, 44) = 20.0, *p* < 0.001] as well as a significant interaction [*F*(8, 88) = 4.9, *p* < 0.01]. Pair-wise comparisons (with corrected alpha level = 0.0033) for bins one to four and the final bin (15) in the PosVF/Deadaptation phase revealed that both Noise and Control were significantly different to Compression for the first two Bins [Min: *F*(2, 22) = 10.32, *p* < 0.001] with both having greater aftereffects. There were no differences between either Noise or Control compared to Compression for the remaining Bins [Max: *F*(2, 22) = 2.01, *p* = 0.16] and no difference between Noise and Control for any Bin [Max: *F*(2, 22) = 0.46, *p* = 0.50]. In summary, Noise and Control showed typical aftereffects, rapidly decaying to baseline whereas Compression exhibited no aftereffects, being at baseline throughout.

## Discussion

**Figure 3:**
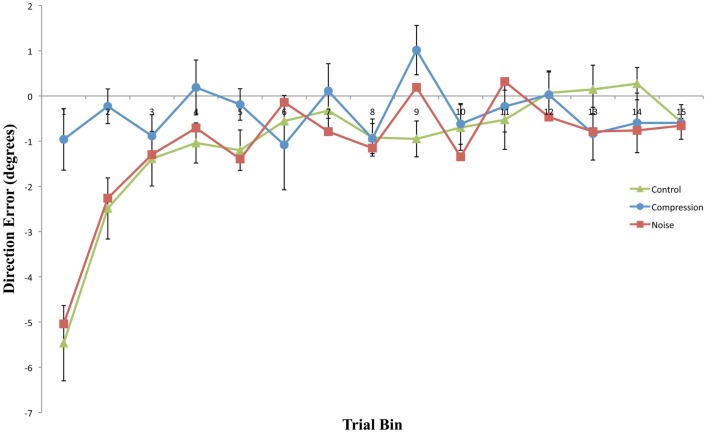
**Mean directional pointing error (with SE bars) for each two-trial bin in the Post-Adaptation phase for all three conditions**. Negative values indicate a leftward error in the direction opposite to the prism displacement.

**Figure 4:**
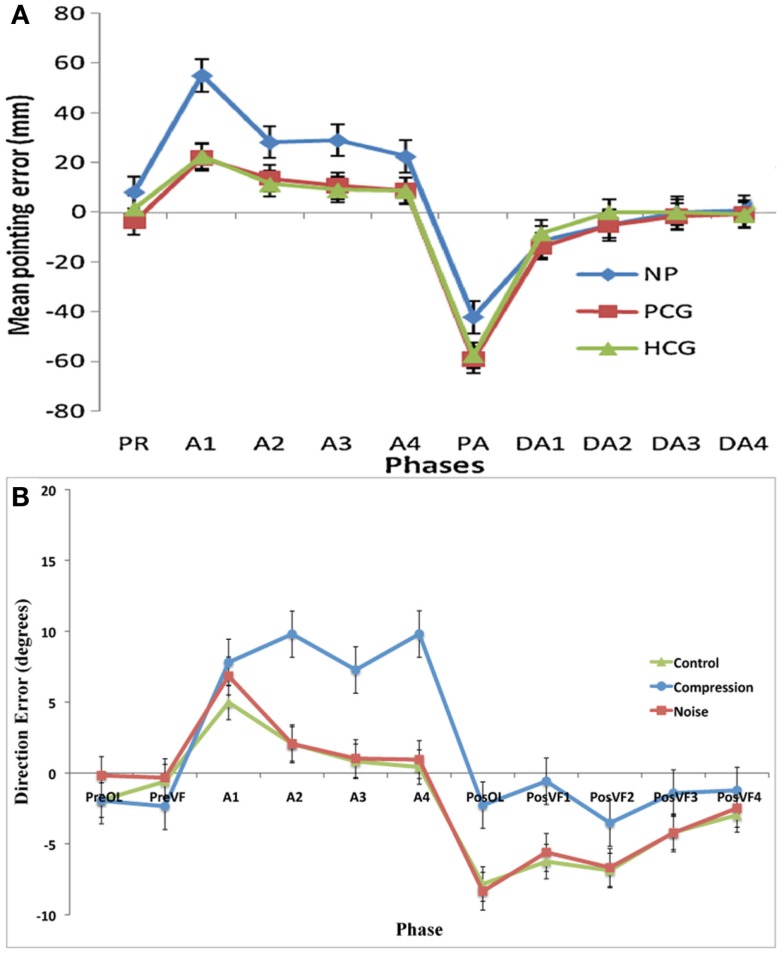
**(A)** Data adapted from Aimola et al. (2012) showing the mean pointing error in millimeter (with SE bars) in each group across five phases: PR, Pre-adapt; A, adaptation; DA, Deadaptation or aftereffect; NP, neglect patients; PCG, patient control group; HCG, healthy control group; **(B)** Current data showing the mean pointing error (with SE) in degrees for the first trials in each phase: PreOL, pre-open loop; PreVF, pre-visual feedback; A (Prism-Adaptation), PosOL; post-open loop; PosVF, Post-visual feedback/deadaptation. Data from Aimola et al. show the means of no visual feedback trials from each block of adaptation.

This experiment was designed to assess whether noisy or compressed visuo-motor environments were able to model the pattern of prism adaption effects observed in neglect patients. With the introduction of noise, both adaptation and aftereffects were indistinguishable from standard PA in healthy controls with both conditions showing an initial rightward shift before returning to baseline accuracy followed by an aftereffect that rapidly decayed (Figures 2 and 3). The introduction of spatial compression, on the other hand, impaired adaptation, and reduced aftereffects in a manner similar to that observed by Aimola et al. (2012) (see Figure 4).

In the compression condition, participants failed to adapt to the prismatic displacement even after 40 trials. Although the actual reaching error was similar to that observed during early trials in the noise and control conditions, the perceived error would have been half that. That is, an error of 6°, large enough to stimulate strategic correction under normal adaptation conditions, would only have been perceived as being an error of 3°, equivalent to a distance of about 1 cm, and potentially below the threshold for detection as an externally generated error. This evidence supports the idea that without conscious registration of the prism-induced perturbations, SC cannot occur.

It should be noted that reducing the perceived 6° error by half is not quite the same as wearing half-strength (3°) prism goggles. Six degree prisms would have perturbed the target by 6°, required a 6° rotation of the eyes (although, see Newport et al., 2009, for a discussion of why this might not be important) and induced a concomitant actual and perceived directional error. In contrast, with 3° prisms target displacement, eye rotation, and directional error would all have been smaller. In the compression condition, compared to 3° displacing prisms, only the perceived error was smaller.

Compression-modeled neglect did not significantly increase the magnitude or longevity of aftereffects relative to the control condition. Indeed, aftereffects were entirely absent both with and without visual feedback of the reaching limb following removal of the prism goggles. In contrast, both Noise and Control post-adaptation reaches displayed similar immediate, but short-lived, aftereffects. As would be expected, these aftereffects were larger in the PosOL phase than in the equivalent visual feedback phase, demonstrating the rapid and normal use of visual feedback in the reduction of prism-induced aftereffects.

It is evident that incomplete SC in compression-modeled neglect does not necessarily lead to larger or longer-term spatial recalibration as reflected by aftereffects. These results closely mimic those of Aimola et al. (2012) (Figure 4), and are contradictory to predictions made by Redding and Wallace (2006) and Michel et al. (2007) that impaired SC in neglect leads to a greater dependency on SR mechanisms and subsequently greater aftereffects. It should be noted, however, that in the current experiment aftereffects were not merely reduced; they were completely absent. This result was unexpected and it would appear that the failure to detect an error at a conscious level (as evidenced by the lack of strategic correction) was mirrored by a failure to detect an error at a lower level. While it is thought that strategic correction helps to promote SR (Redding and Wallace, 1996), a failure of strategic correction can lead to excessive realignment and abnormally large aftereffects (Newport and Jackson, 2006). In this case, however, there was neither correction nor realignment. It is possible that the error in the current study was too small to require the motor system to correct or that, given the size of the error, not enough trials were completed in order to produce noticeable aftereffects.

Regardless of whether the spatial compression applied here is an accurate representation of the visuo-motor experience in neglect, dynamically altering multisensory interactions using virtual reality could provide a promising avenue for rehabilitative research. Spatial representations are the result of dynamic remapping processes determined by multisensory input. PA creates an additional rightward bias to that already present in neglect, and patient’s recalibration for this seems to trigger subsequent recalibration of their task-work space position. Redding and Wallace (2006), however, speculate that PA is ineffective in recalibrating *size* of the work space, and that this may result from a compressed spatial representation. Thus, manipulation of the visual workspace and the subsequent compensatory visuo-motor adjustment to this may theoretically enable neglect patients to correct the size of the task-work space as well as the spatial recalibration. Indeed, with the current system it would be possible to create a visual workspace based upon the Oppel–Kundt illusion which has been shown to modulate both neglect and healthy control performance on visuo-spatial tasks (Savazzi et al., 2007, 2012; Pia et al., 2012). Future research could therefore focus on determining the characteristics of compressed distortion in individual patients and assess whether dynamically resizing the visual workspace in accordance with that distortion could be more beneficial in rehabilitating neglect than standard, rigid, prism goggles.

In summary, compression-modeled neglect successfully impairs SC in PA, replicating the results found by Aimola et al. (2012) in neglect patients. Alongside previous research and theories for neglect syndrome, these results suggest that spatial representations primarily involved in visuo-spatial behavior is compressed in neglect and that investigations that manipulate anisotropic distortions of the visual workspace may be a fruitful avenue of research for rehabilitation.

## Conflict of Interest Statement

The authors declare that the research was conducted in the absence of any commercial or financial relationships that could be construed as a potential conflict of interest.
